# Tumor-derived exosomal HOTAIRM1 regulates SPON2 in CAFs to promote progression of lung adenocarcinoma

**DOI:** 10.1007/s12672-022-00553-7

**Published:** 2022-09-24

**Authors:** Zhipeng Chen, Chengyu Bian, Jingjing Huang, Xiang Li, Liang Chen, Xueying Xie, Yang Xia, Rong Yin, Jun Wang

**Affiliations:** 1 Department of Thoracic Surgery, Jiangsu Province People’s Hospital and the First Affiliated Hospital of Nanjing Medical University, Nanjing, Jiangsu 210029 China; 2 Department of Thoracic Surgery, Shanghai Pulmonary Hospital, Tongji University School of Medicine, Shanghai, China; 3 State Key Laboratory of Bioelectronics, School of Biological Sciences and Medical Engineering, Southeast University, Nanjing, China; 4 Department of Thoracic Surgery, Jiangsu Key Laboratory of Molecular and Translational Cancer Research, the Affiliated Cancer Hospital of Nanjing Medical University and Jiangsu Cancer Hospital and Jiangsu Institute of Cancer Research, Nanjing, Jiangsu 210000 China

**Keywords:** Lung adenocarcinoma, Tumour microenvironment, CAFs, Exosomes, SPON2

## Abstract

**Objective:**

SPON2 is one of the extracellular matrix proteins, which is closely related to the progression of a variety of tumors including non-small cell lung cancer (NSCLC), but its upstream regulation mechanism remains unclear. Our research aims to find the specific regulatory pathway of SPON2 by exploring the potential crosstalk between tumor cells and cancer-associated fibroblasts (CAFs) in tumor microenvironment (TME) of NSCLC.

**Methods:**

We analyzed T1 lung adenocarcinoma samples from TCGA and screened extracellular matrix proteins that indicate poor prognosis. Expression level of SPON2 was verified by qPCR in clinical samples. The exosomes of NSCLC cell supernatant were extracted and identified by nanoparticle tracking analysis (NTA) and transmission electron microscope, western blots. The exosomes and CAFs were co-cultured, and cell migration and Matrigel invasion assay were used to evaluate the effect of CAFs on the migration and invasion of NSCLC cells. The interaction between LncRNA and miRNA was verified by Targetscan prediction, luciferase reporter assay, and RNA binding protein immunoprecipitation (RIP).

**Results:**

We found that the expression of SPON2 was up-regulated in clinical T1a stage NSCLC patients. The expression of lnc HOTAIRM1 (HOTAIRM1) in exosomes secreted by NSCLC tissues increased. After exosomal HOTAIRM1 entered CAFs, HOTAIRM1 can adsorb miR-328-5p to up-regulate the expression of SPON2 in CAFs. Up-regulation of SPON2 in CAFs could promote the migration and invasion of NSCLC cells.

**Conclusion:**

Tumor-derived exosomal HOTAIRM1 can transfer into CAFs and competitively adsorb miR-328-5p, and regulate the SPON2 expression of CAFs cells, ultimately promote the progression of NSCLC. The discovery of this regulatory pathway can provide a new potential therapeutic target for the diagnosis and treatment of NSCLC.

**Supplementary Information:**

The online version contains supplementary material available at 10.1007/s12672-022-00553-7.

## Introduction

Lung cancer is still one of the tumors with high morbidity and mortality [1]. The vigorous implementation of the lung cancer screening program has significantly increased the detection rate of early lung cancer [2]. However, even in the early stage of lung cancer, tumor heterogeneity also leads to the risk of progression and migration, which is the main factor of poor prognosis of lung adenocarcinoma [3]. Therefore, it is of great clinical significance to study the triggering mechanism of metastasis in early lung cancer.

In recent years, the role of TME in the research of tumor cell differentiation, proliferation, invasion and metastasis and drug resistance has attracted more and more attention [4–6]. According to the “seed & soil” theory, the crosstalk between microenvironment stromal cells and tumor cells plays an important regulatory role in the progression of tumor invasion [7, 8]. CAFs is one of the fundamental cell components with the highest proportion in tumor matrix, which has a strong ability of extracellular matrix remodeling, and promotes tumor progression by inducing stromal cell fibrosis and increasing matrix tension [9, 10]. The ability of CAFs to reshape the microenvironment is not a unique phenomenon, and as with other stromal cells, interactions between tumor cells and matrix cells play an important role [11]. Among them, for example, the delivery of small molecules through exosome pathway has attracted increasing attention of researchers.

Exosomes are vesicles with a diameter of 40 to 160 nm (an average of 100 nm) derived from the interior of cells. They contain a large number of substances, such as DNA, RNA, lipids, metabolites, cytoplasmic and cell surface proteins, which play various vital functions in intercellular communication and modulate biological behavior in downstream cells [12, 13]. It has been reported that exosomes derived from NSCLC can activate CAFs cells to secrete extracellular matrix, but the specific mechanism is not completely clear [14]. SPON2 is one of the extracellular matrix proteins. Physiologically, it can bind to bacteria and participate in the phagocytosis of bacteria by macrophages. It is a unique pattern recognition molecule of microbial pathogens in ECM and crucial in the initiation of innate immune response [15].In early studies, overexpression of SPON2 was confirmed to be an independent prognostic biomarker of lung adenocarcinoma. However, the specific mechanism for its overexpression is still unclear [16]. Our preliminary study found that the increased SPON2 expression level in early NSCLC patients was associated with poor prognosis, and an oncogenic long non-coding RNA (LncRNA), HOTAIRM1 in exosomes secreted from NSCLC cells may be associated with regulating SPON2 expression in CAFs. HOTAIRM1 can regulate neuronal differentiation and promote osteogenesis in physiological state, and inhibit T-cell depletion in late sepsis, so as to reduce lung injury and improve the survival rate.HOTAIRM1 has been proved to promote the progression of lung adenocarcinoma [17, 18], and its positive correlation with SPON2 expression attracts our interest, which means that it may also have other cancer promoting pathways.However, the exact mechanism is unclear. The purpose of this study was to explore the role and the exact mechanism of exsomal LncRNA in crosstalk between tumor cells and CAFs in tumor microenvironment.

## Materials and methods

### Analysis of the correlation between SPON2 and prognosis of lung adenocarcinoma in stage T1a in the TCGA database

Lung adenocarcinoma samples at stage T1a in TCGA were downloaded and extracellular matrix proteins associated with poor prognosis [19] in these samples were analyzed using the R software.The cell subpopulations related to ECM were analyzed by deconvolution calculation.The data of GSE12428 and GSE11969 in GEO database were analyzed by survival analysis database LOGpc [20].

### Patient and tissue samples

Clinical specimens were collected from 10 patients underwent operation in Jiangsu Provincial People’s Hospital. The study was approved by the ethics committee of Jiangsu Provincial People’s Hospital (No. 2019-SR-266), and the patient was informed and signed consent forms. The research was conducted in accordance with the Declaration of Helsinki (as revised in 2013).

### Cell lines and cell culture

A549 and H1299 were purchased from Chinese Academy of Sciences, Shanghai Institute of Biochemistry and Cell Biology (Shanghai, China). A549/Luc cells (A549 cells stably expressing luciferase) were constructed by Synthgene (Nanjing, China). A549 was cultured with DMEM (Dulbecco’s Modified Eagle’s Medium) (Gibco, Rockville, USA) and H1299 was cultured with Roswell Park Memorial Institute (RPMI) 1640 medium supplemented with 10% FBS (fetal bovine serum) (Gibco, Rockville, USA) and 100 µg/mL streptomycin and penicillin (Gibco, Rockville, USA) in a humidified atmosphere at 37 ℃. CAFs were isolated from surgical specimens diagnosed as T1 stage lung adenocarcinoma by pathology. Details of these patients are in the “Information About CAFs from Patients' in the Additional file 1. Tumor tissue was dissected and digested with trypsin and collagenase to obtain single cell suspension for culture, and then maintained in DMEM high glucose culture medium (including 10% FBS, Gibco).GW4869(HY-19,363, MedChemExpress, China) is used to inhibit the release of exosomes.

### Cell transfection

miR-328-5p inhibitor, mimic and SPON2 and HOTAIRM1 expression plasmid were synthesized from Nanjing Relgene biological company(Inhibitor is an RNA that can reduce miRNA expression through base complementation, and mimic is an RNA with the same sequence as miRNA that can improve expression); HOTAIRM1 overexpression lentivirus was synthesized from Shanghai Hanbio biological company, and HOTAIRM1 siRNA was purchased from Guangzhou RiboBio biological company. Lipofectamine RNA IMAX was used to transfect siRNA, miR-328-5p inhibitor and mimic, and lip3000 and P300 (Invitrogen, USA, L3000-015) were used to transfect plasmids overexpressing SPON2.In tumor cells, we used lentivirus transduction for HOTAIRM1 overexpression, while for miR-328-5p knockdown and overexpression, both tumor cells and CAFs used mimics and inhibitors. Since CAFs cannot be stably subcultured, siRNA and plasmid are used for knockdown and silencing, whether HOTAIRM1 or SPON2. After screening the best viral titer and puromycin concentration overexpressing HOTAIRM1, the cell transduction process was carried out according to the specification. Puromycin (ST551, Beyotime, China) is used to screen and maintain stable lentivirus strains.

### 5 Western blot analysis

Western blot analysis was performed as described in our previous study [11]. The protein concentration was quantified by the BCA protein quantification Kit(P0012, Beyotime, China), refer to the instructions of the kit for experimental operation. The primary antibodies in this study were purchased from Abcam: rabbit anti-CD9(ab236630, 1:500), TSG101(ab30871, 1:500), SPON2(ab171955, 1:1,000), ago2(ab186733, 1:500) and glyceraldehyde-3-phosphate dehydrogenase(GAPDH)(ab8245, 1:5,000). The horseradish peroxidase labeled goat anti-rabbit IgG antibody (ab205718, 1:10,000) and goat anti-mouse (ab6789, 1:10,000) were available as the secondary antibodies. Image J software was used to quantify each protein band.

### Extraction and identification of exosomes

The lung adenocarcinoma cells were cultured in DMEM or RPMI 1640 supplemented with 10% FBS without exosomes for 24 h, and the culture supernatant was collected. Exosomes were isolated and purified from conditioned media of lung adenocarcinoma cells according to the standard isolation method of exosome with ultracentrifugation. Bicinchoninic Acid Assay (BCA) method was carried out to measure the total protein concentration of isolated exosomes, which was completed by a professional kit (P0012, Beyotime, China). Transmission electron microscopy (Hitachi ht7700, Tokyo, Japan) was used to verify the morphology of the isolated exosomes. Nanoparticle tracking analysis(NTA) was completed by Shanghai XP Biomed Ltd. and exosome characteristic proteins CD9 and TSG101 were identified by Westerns Blot. Dio (green) and DIL (red) are used to label exosome membrane and CAFs cell membrane to verify that exosomes are ingested by CAFs. Exosomes were ingested by CAFs and photographed by confocal microscope.

### RNA extraction and quantitative real-time PCR analysis

The extraction and reverse transcription of total RNA were performed according to the previous report [11]. The expression levels of SPON2, HOTAIRM1 and miR-328-5p were analyzed by quantitative real-time PCR with the glyceraldehyde-3-phosphate dehydrogenase (GAPDH) gene or U6 as a standard control. GAPDH was served as reference for the total RNA of PCR cells, U6 for the miRNA, and λ polyA (Code No. 3789, takara, Japan) for the exosome LncRNA. Primers of SPON2, HOTAIRM1, miR-328-5p and U6 were as Table 1. These primers were synthesized and purified by RiboBio (Guangzhou, China).

**Table 1:** The sequences of oligonucleotides and primers

Primer	Forward primer	Reverse primer
HOTAIRM1	AGGGGGTTGAAATGTGGGTGA	CTTGAAAGTGGAGAAATAAAGTGCC
SPON2	TCCTTTAACACGCGAGGCTT	TCTACCCCGTTCCTCATCGT
miR-328-5p	GGGGGCAGGAGGGGC	GTCGTATCCAGTGCAGGGTCCGAGGTATTCGCACTGGATACGACCCCTGA
U6	TCGGCAGCACATATACTAA	CGCTTCACGAATTTGCGTGT
GAPDH	GACCTCAACTACATGGTT	AACCATGTAGTTGAGG

### Cell migration and invasion assays

The cell invasion and migration assays were performed by 24-well Transwell cell culture chambers with 8-µm sized pores with or without precoated Matrigel (BD Biosciences, San Jose, CA, USA). Same as before [11], A549 cells at a density of 2 × 10^4^ cells/mL, were resuspended with 200µL DMEM medium (serum-free) and seeded into the upper chamber, while the lower chamber was placed with CAFs transfected with different plasmids, cultured with 600 µL DMEM medium (10% FBS, Gibco, Rockville, USA). After 24 h, the cells left in the upper chamber were removed. The cells which invaded or migrated were fixed with Paraformaldehyde, 4% (Solarbio, China) for 30 min, and stained with 0.1% crystal violet solution for 15 min, and then photographed under the inverted microscope.

### Immunofluorescence assay and fluorescence in situ hybridization (FISH)

Immunofluorescence assay was performed as described in our previous study [11]. Cy3 labeled HOTAIRM1 and fish Kit (Ruibo biology, Guangzhou, China) were used in accordance with the manufacturer’s instructions. Images were captured using FV10i confocal microscope (Olympus, Japan).

### Immunoprecipitation assay

RIP kit was purchased from Magna(Cat. #17-700, USA); The total RNA of CAFs was obtained on the specification. After washing, immunoprecipitated RNA was isolated and qPCR was performed.

### Luciferase reporter gene

Luciferase reporter gene detection kit was purchased from Promega (the USA). HOTAIRM1 or SPON2 3’UTR fragments covering the binding sites of wild-type (WT) or mutant (MUT) miR-328-5p were prepared to generate pmirglo-HOTAIRM1-wt/mut and pmirglo-SPON2 3’utr-wt. They were co-transfected into CAFs cells with NC (A meaningless RNA as a control for miR-328-5p) or miR-328-5p mimic for 48 h.

### Statistical analysis

All statistical analyses in this study were performed using Graphpad prism 8.0 software. Mann-Whitney test was used to compare two groups of clinical patients’ genes expression. Kaplan-Meier analysis was performed for survival analysis. Statistical analysis was performed using the Student’s two-tailed t-test and one-way analysis of variance (ANOVA). Asterisk indicates significant difference (*P < 0.05; **P < 0.01; ***P < 0.001).

## Result

### 1 SPON2 is up-regulated in T1 stage lung adenocarcinoma

**Fig. 1:**
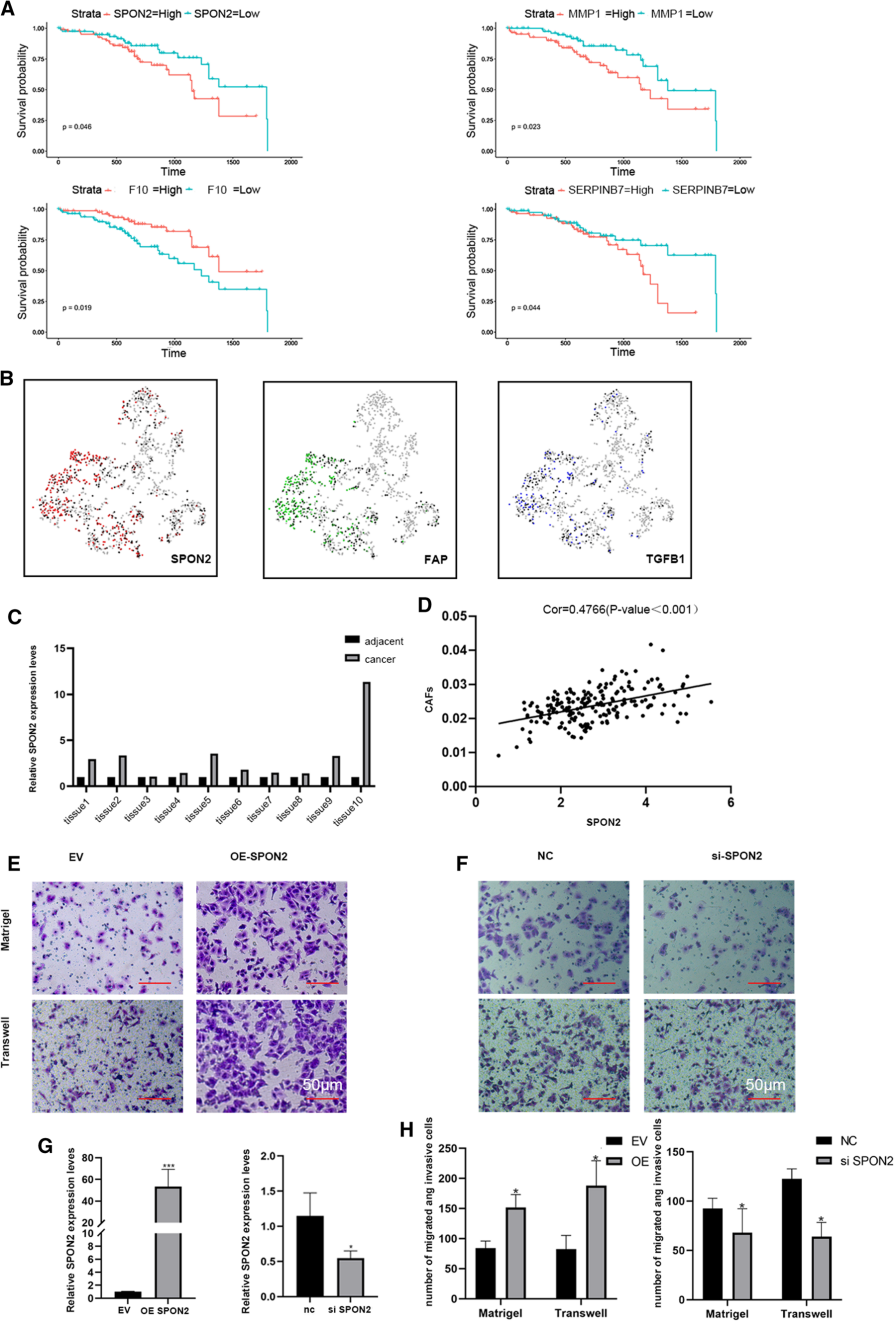
High expression of SPON2 in CAFs affects the prognosis of lung adenocarcinoma. **A** TCGA database analyzed the survival curve of up-regulated ECM in NSCLC. **B** Single cell sequencing data were used to analyze the correlation between SPON2, TGFB1 and FAP. **C** The expression of SPON2 in tumor and adjacent normal tissues was detected by qPCR. **D** The correlation between SPON2 and CAFs was analyzed by deconvolution method. (**E**–**H**) The migration and invasion abilities were measured by transwell assay in A549 cells (200× magnification). Scale bar = 100 μm. **G** Efficiency of SPON2 knockdown and overexpression

Analysis of 191 cases of T1 stage lung adenocarcinoma samples from the TCGA database revealed that the high expression of four tumor related extracellular matrix proteins was associated with poor clinical prognosis (Fig. 1A). The analysis of LOGpc further confirmed that the high expression of SPON2 was associated with the poor prognosis of early lung adenocarcinoma (Additional file 3: Fig. S1). Using R software to analyze the data of SPON2 and Lambrechts et al., we found that the calculation results suggest that SPON2 coincides with TGFB1 and FAP, which means that SPON2 is related to them [21]. (Fig. 1B). Then, qPCR was performed to verify the total RNAs extracted from ten pairs of clinical samples resected during surgery, and the results showed that the expression of SPON2 in tumor tissues was significantly higher than that of the adjacent normal tissues (Fig. 1C). Interestingly, the deconvolution calculation results of TCGA database revealed that SPON2 expression is closely related to infiltration of CAFs (Fig. 1D).

### High expression of SPON2 in CAFs promoted the migration and invasion of NSCLC

Two NSCLC cell lines, A549 and H1299, were spread in Transwell and Matrigel chambers respectively, and co-cultured with SPON2-overexpressing CAFs for 24 and 48 h. Figure 1G shows the efficiency of SPON2 gene knockout and overexpression.The results showed that overexpression of SPON2 in CAFs significantly promoted migration and invasion of NSCLC compared with the EV group (Fig. 1E). Inhibition of SPON2 in CAFs reduced the aggressiveness of co-cultured tumor cells compared with the NC group (Fig. 1F). Figure 1G shows the transfection efficiency of overexpression and knockdown of SPON2.The migration and invasion function of H1299 is shown in Additional file 3: Fig. S2A–C.

### 3 Tumor derived exosomal HOTAIRM1 up-regulates the expression of SPON2 in CAFs

**Fig. 2:**
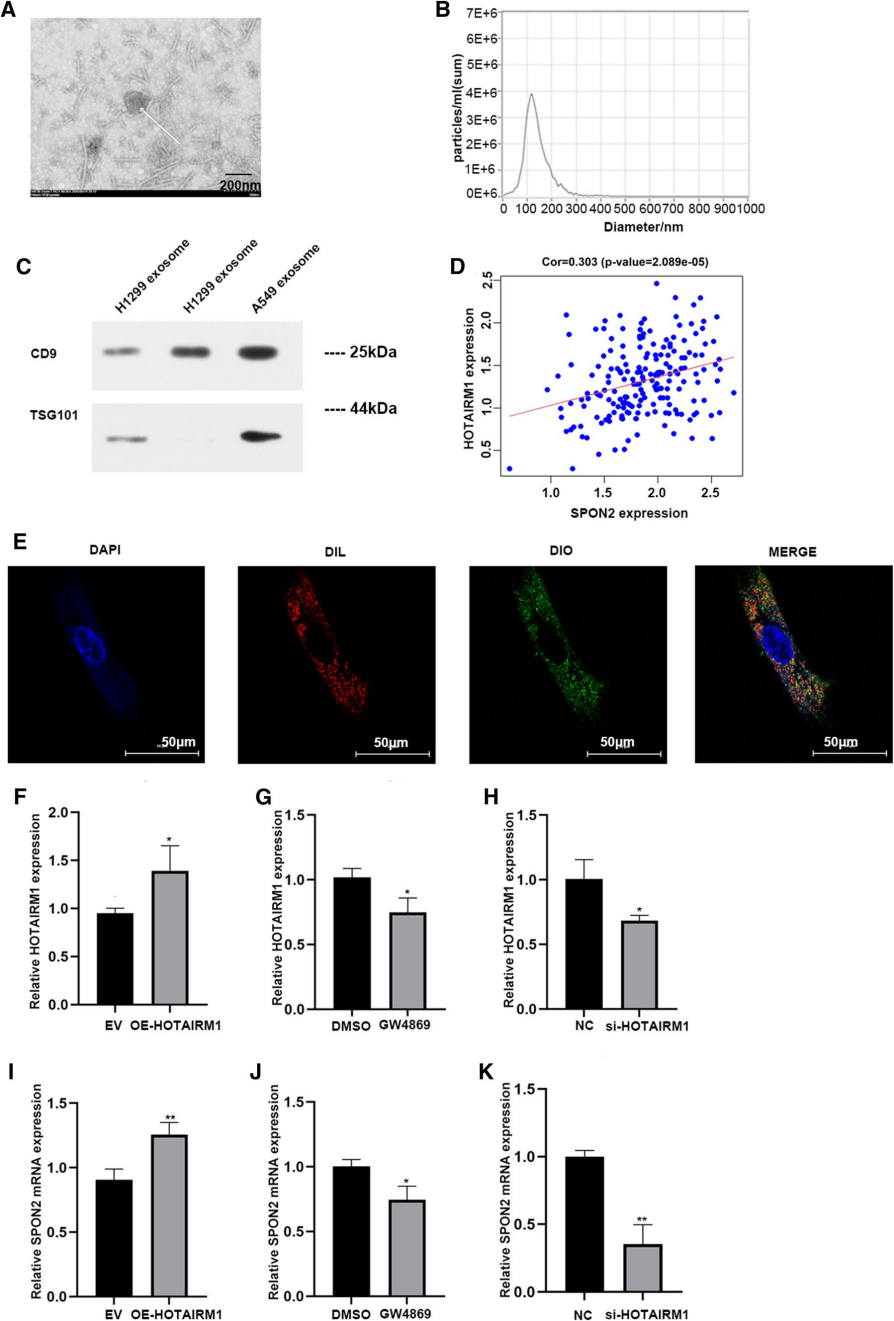
Exosomes secreted by A549, H1299 regulate the expression of SPON2 in CAFs. **A** The structure of exosome was identified by transmission electron microscope (40.0k× magnification). Scale bar = 200 nm. **B** The size of exosome was detected by nanoparticle tracking analysis. **C** The protein levels of exosomal markers TSG101, CD9 were analyzed by western blot. **D** Correlation analysis between HOTAIRM1 and SPON2 (Cor = 0.303, p-value = 2.089e−05). **E** The fluorescence signal of CAFs cells babeled by DIL co-cultured with A549 exosomes labeled by DIO was detected (400× magnification). Scale bar = 50 μm. **F**–**H** After co-cultured with lung cancer cells were knocked-down, overexpressed of HOTAIRM1 or transfected by GW4869, the expression of HOTAIRM1 in CAFs. **I**–**K** After co-cultured with lung cancer cells were knocked-down, overexpressed of HOTAIRM1 or transfected by GW4869, the expression of SPON2 in CAFs

In order to study the interaction between tumor cells and CAFs, exosomes were collected from the supernatant of A549 and H1299 cells, and the isolated exosomes were detected by electron microscopy. A typical exosome is marked with a white arrow in Fig. 2A (scale, 200 nm). Further analysis of the purified particles using nanoparticle tracking analysis (NTA) showed particle sizes in the 50–200 nm range (Fig. 2B). Western blot was used to detect the expression of CD9 and TSG101 in exosomes (Fig. 2C) and the original bands are shown in Additional file 3: Fig. S3. We sequenced the lncRNA chip in the exosomes of A549 and orrelation analysis showed that HOTAIRM1 was positively correlated with SPON2 (Fig. 2D). Exosomes of A549 cells were labeled with DiO and co-cultured with CAFs labeled by DiL for 24 h, and the nucleus of CAFs was dyed blue with DAPI. We confirmed that exosomes from A549 cells were taken up by CAFs by confocal microscopy (Fig. 2E). The exosomes from H1299 cells were taken up by CAFs by confocal microscopy is shown in Additional file 3: Fig. S2D. Subsequently, A549 overexpressing HOTAIEM1 and control group were co-cultured with CAFs, respectively, and qPCR results showed the expression of SPON2 was up-regulated in CAFs in the HOTAIRM1 overexpression group (Fig. 2F, I), which was consistent with the results of previous analysis (Fig. 2D), and the expression of exosomal HOTAIRM1 after overexpression of HOTAIRM1 in A549 cells, is shown in Additional file 3: Fig. S4. In order to confirm that SPON2 in CAFs is regulated by exosomes rather than other pathways, we cultured tumor cells in culture medium containing GW4869 (a reagent that can inhibit exosome release [22]) and co-cultured them with CAFs for 48 h. The results showed that the expression of HOTAIRM1 in CAFs decreased after inhibition of A549 exosome secretion (Fig. 2G, J). Besides, we also knocked down HOTAIRM1 of tumor cells and co-cultured with CAFs. Subsequently, we observed that SPON2 expression in CAFs decreased synchronously (Fig. 2H–K). These synergistic changes supported that SPON2 in CAFs could be regulated by HOTAIRM1 from tumor cell exosomes.

### HOTAIRM1 regulates the mRNA level of SPON2 by competing with miR-328-5p

**Fig. 3:**
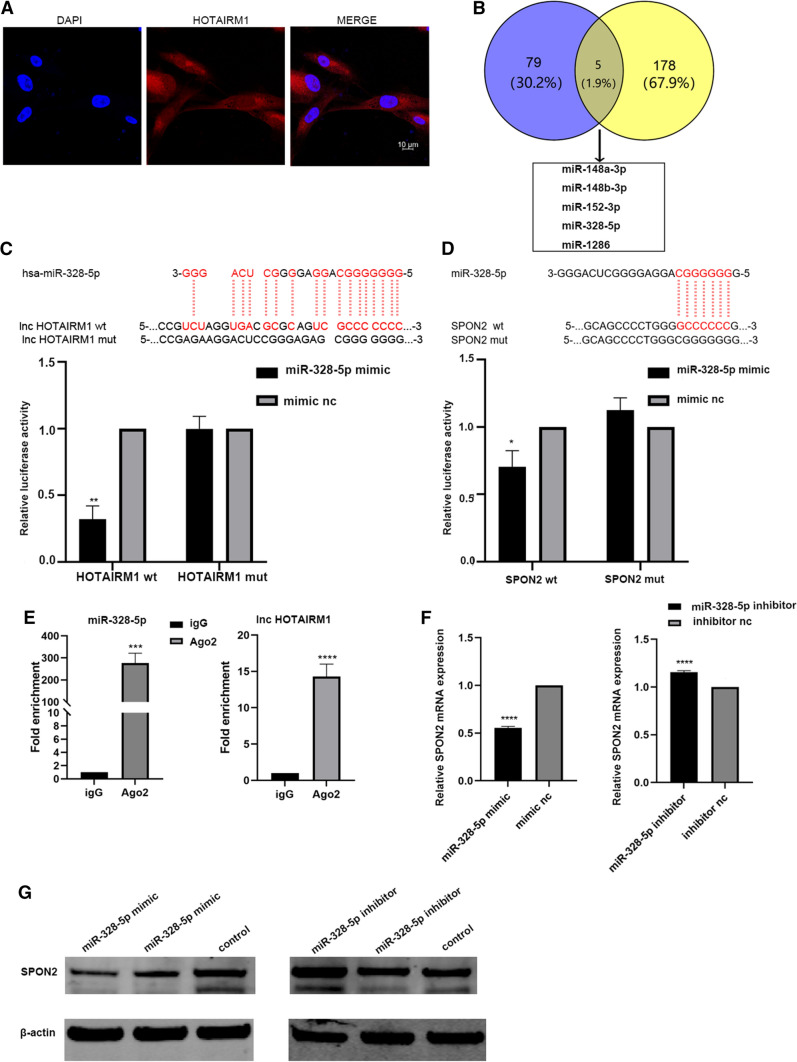
HOTAIRM1 exerts its biological function by competitive adsorption of miR-328-5p. **A** Fluorescence in situ hybridization(FISH) detects HOTAIRM1 position. **B** Online software predicts miRNAs and their intersections associated with HOTAIRM1 and SPON2. **C**, **D** Luciferase reporter gene detected the binding sites of miR-328-5p with HOTAIRM1 and SPON2. **E** RIP experiment verified that HOTAIRM1 and miR-328-5p were involved in the formation of RISC complex. **F**, **G** miR-328-5p inhibits SPON2 expression was detected to inhibit the expression of SPON2.

**Fig. 4:**
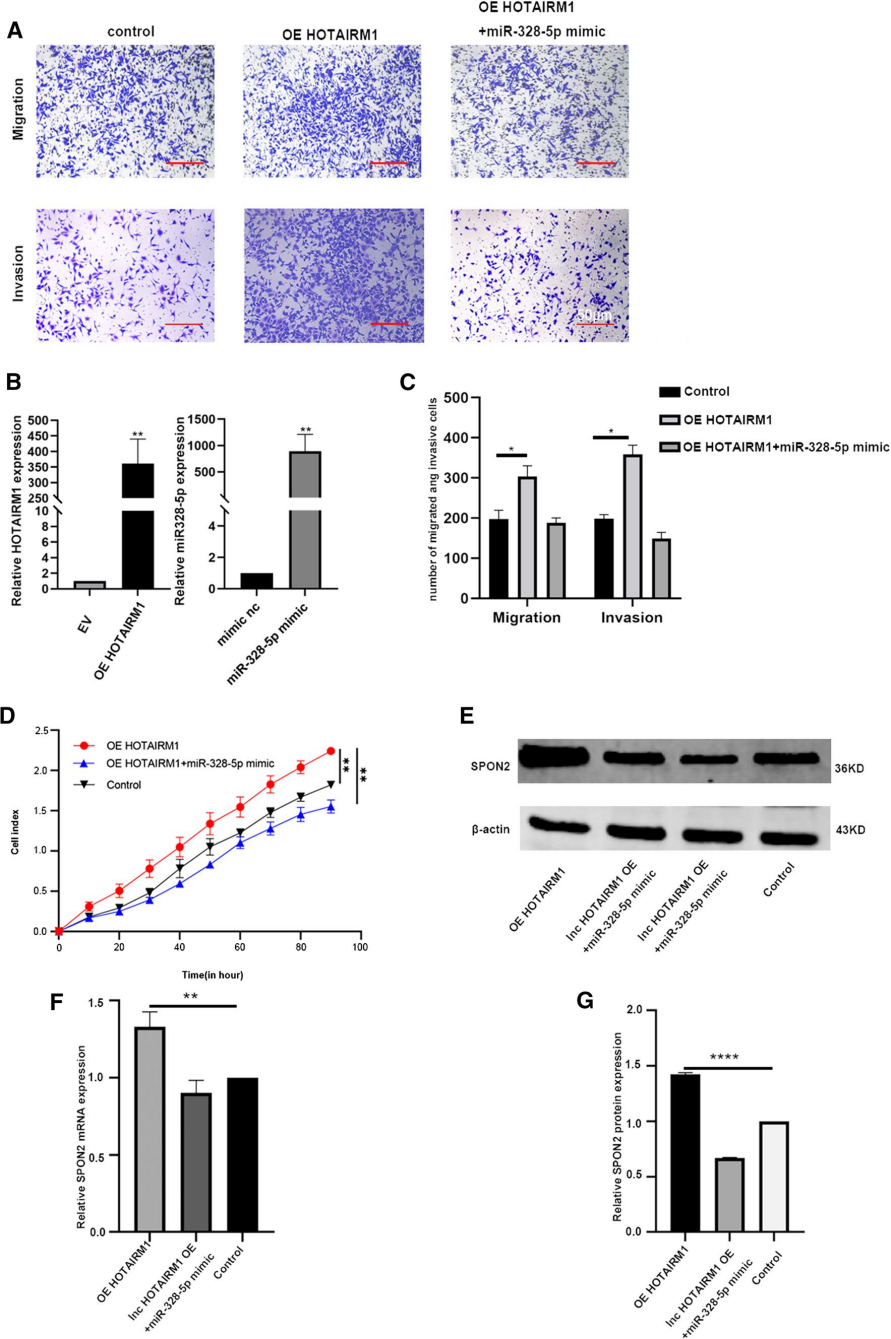
miR-328-5p promotes the migration and invasion of A549 cells by regulating the expression of SPON2 in CAFs. **A** The migration and invasion abilities were measured by Transwell and Matrigel assay in A549 cells (200× magnification). Scale bar = 50 μm. **B** PCR verifies the efficiency of overexpression of HOTAIRM1 and miR-328-5p simulants. **C** RTCA curve shows the invasion of A549. **D**, **F** The protein expression levels of SPON2 were analyzed by western blot and qPCR in A549 cells. **E** The mRNA expression levels of SPON2 were analyzed by western blot and qPCR in A549 cells. Error bars represent standard deviations and asterisks show significant differences from corresponding control according to Student’s t-test (*P < 0.05, **P < 0.01, ***P < 0.001)

In order to explore the specific regulating mechanism of HOTAIRM1 to SPON2, the localization of HOTAIRM1 in CAFs was firstly confirmed by fluorescence in situ hybridization (FISH) experiment. HOTAIRM1 was distributed in the cytoplasm and nucleus, and mainly in the cytoplasm (Fig. 3A). In recent years, many studies reported that LncRNA in cytoplasm plays a biological role mainly through competitive combination with miRNA. We analyzed the possible pathway of HOTAIRM1 by online software, and also found that HOTAIRM1 could work through the mechanism of competing endogenous RNAs (ceRNA). The miRNAs predicted to bind to HOTAIRM1 and SPON2 were crossed by online software [23], and finally five miRNAs were selected, which can bind SPON2 and HOTAIRM1 at the same time in the prediction results (Fig. 3B), software prediction results in “Predicted miRNA Binding to SPON2 and HOTAIRM1” (Additional file 2). The wild type and mutant double luciferase reporter gene plasmids were constructed at the 3 ‘UTR region binding sites of miR-328-5p, HOTAIRM1 and SPON2, respectively (Fig. 4C, D). Further verification by luciferase reporter gene experiment showed that only miR-328-5p could bind to SPON2. Compared with the control group, the luciferase activity in the co-transfection group of wild-type luciferase plasmid and miR-328-5p simulants decreased significantly, which proved that miR-328-5p could bind to the 3’UTR region of HOTAIRM1 and SPON2 (Fig. 3C, D). The ago2 RIP experiment results showed that HOTAIRM1 could sponge-adsorb miR-328-5p (Fig. 3E). Subsequently, miR-328-5p simulants were transfected into CAFs. The qPCR results showed that the expression of SPON2 decreased significantly, which proved that miR-328-5p could inhibit the expression of SPON2 (Fig. 3F, G) (The original WB band is shown in Additional file 3: Figs. S5, S6).

### The depletion of miR-328-5p eliminated the effect of HOTAIRM1 on the up-regulation of SPON2 expression

.

Rescue experiments were carried out to confirm the role of HOTAIRM1/miR-328-5p/SPON2 pathway in promoting the migration and invasion of non-small cell lung cancer. CAFs cotransfected with the overexpressing HOTAIRM1 plasmid, miR-328-5p mimics were co-cultured with A549 for 24 h. After overexpression of HOTAIRM1, the invasion and migration of tumor cells were significantly improved, but after transfection of miR-328-5p simulant, the effect of HOTAIRM1 was weakened (Fig. 4A, C), and The overexpression efficiency of HOTAIRM1 and miR-328-5p was also verified (Fig. 4B). What’s more,the proliferation curve of A549 was also lower than that of the control group (Fig. 4D).The expression levels of SPON2 protein and RNA also increased after overexpression of HOTAIRM1, while miR-328-5p weakened the effect of HOTAIRM1 (Fig. 4E–G). The migration and invasion function of H1299 is shown in Additional file 3: Fig. S2E and the original WB band is shown in Additional file 3: Fig. S7.

## Discussion

In recent years, more and more studies have found that extracellular matrix (ECM) plays very important regulatory roles in tumor genesis and development [24, 25]. Abnormalities of extracellular matrix, such as tissue interruption and changes in basic components or morphology of extracellular matrix, can modulate the behavior of stromal cells and promote tumor-related angiogenesis and inflammatory response, which are related to the occurrence and metastasis of cancer [19]. A number of studies have reported that tumor cells can interact with stromal cells to dynamically regulate tumor microenvironment (TME) and eventually induce apoptosis or progression of tumor cells [26–31]. In these crosstalks, changes of proteins expressed by stromal cells are important link [32]. However, due to the extremely complex regulatory process of these proteins in the matrix environment, the molecular mechanism of matrix proteins involved in lung cancer progression is not fully understood.

In this study, an extracellular matrix protein SPON2 was found to be highly expressed in T1 stage lung adenocarcinoma and associated with poor prognosis by analysis of TCGA database. SPON2 (also known as spondin-2) is a highly conserved ECM protein. It is a member of the Mindin-f-Spondin (FS) family of secreted ECM proteins [33] and is widely expressed in spleen and lymph nodes, but not in lung cancer cells [34]. In recent years, SPON2 has been proved to promote cancer progression in many malignant tumors, such as by regulating Notch signaling pathway or activates integrin β1/Pyk2 axis to promote tumor migration and growth and is associated with lymph node metastasis [31, 35–37]. Especially, SHOTARO KURAMITSU et al. found that SPON2 was highly expressed in CAFs, which can increase the proliferation and invasion of gastric cancer cells. More interestingly, it is also associated with peritoneal dissemination in gastric cancer [37]. CAFs is one of the highest cellular components in tumor matrix and has strong ability of remodeling extracellular matrix. It also promotes tumor progression by inducing stromal cell fibrosis and increasing stromal tension [9, 10].

In this study, by deconvolution calculation of T1 stage lung adenocarcinoma in TCGA database, it was found that the expression of SPON2 was closely related to the infiltration of CAFs. Interestingly, it’s contradictory to results of other previous reports that lung cancer cells do not express SPON2 [34], which attracts our interest. What’s more, how CAFs interact with tumor cells and the SPON2 regulation mechanism in CAFs have not been thoroughly studied. Whether tumor cells directly regulate SPON2 expression in CAFs has not been reported. Here we try to explore this phenomenon. It has been recognized that exosome-mediated intercellular communication widely existed between tumor cells and stromal cells [38, 39]. In our recent study, we found exosomes mediated the communication between tumor-associated macrophages (TAMs) and tumor cells and promoted the progression of NSCLC [11]. This also inspired us that tumor cells may regulate SPON2 expression in CAFs through exosome pathway, so as to promote the migration and invasion of tumor cells. Further cell experiments confirmed that exosomes HOTAIRM1 could be absorbed by CAFs, and changes of HOTAIRM1 could lead to corresponding changes of SPON2 expression in CAFs. These results reveal that HOTAIRM1 in exosomes derived from NSCLC cells regulates the expression of SPON2 in CAFs, which provides new evidence for exosome-mediated communication between tumor cells and ECM cells.

HOTAIRM1 is an oncogenic lncRNA, and in recent years, it has been proved to regulate the proliferation and invasiveness of many malignant tumors and affect the prognosis of tumors [40–42].However, some studies have found that HOTAIRM1 played an anti-cancer role in some tumors [43–45] and its specific biological function in lung adenocarcinoma is still unclear. There is an interaction mechanism hypothesis called competitive endogenous RNA (ceRNA) between non coding RNA and RNA (mRNA) with protein coding ability, that is, ncRNA containing miRNA binding sites (such as lncRNA, circRNA, etc.) can release the inhibition of miRNA on target genes by binding with miRNA [46].Given that the common biological mechanism of lncRNA is the ceRNA mechanism, we used bioinformatics methods and molecular experiments for further exploration, and found that after being captured by CAFs, HOTAIRM1 could definitely bind miR-328-5p to regulate SPON2 through the ceRNA mechanism. Although studies have showed that miR-328-5p play a tumor suppressive role in a variety of tumors [47, 48], there are few reports on the function of miR-328-5p in NSCLC. In our study, we found that HOTAIRM1 in lung adenocarcinoma cell exosomes, as upstream of miR-328-5p, can reduce their anti-tumor effects through competitive adsorption of the miRNA. Whether HOTAIRM1 has other biological functions in NSCLC and whether SPON2, as an important cancer-promoting ECM protein, has other regulatory pathways deserve further investigation, and it is now underway in our group.

In our series of experiments, we confirmed that exosomes-mediated communication existed between lung adenocarcinoma cells and stromal CAFs cells. Tumor exosomal HOTAIRM1 can transfer into CAFs and competitively adsorb miR-328-5p through the ceRNA mechanism, and regulate the SPON2 expression of CAFs cells, ultimately promote the progression of NSCLC. This study provides a theoretical basis for migration and invasion of NSCLC from the perspective of tumor microenvironment, and also provides a new idea and target for clinical treatment of NSCLC. Definitive therapy targeting on SPON2 or HOTAIRM1 in tumor microenvironment may improve the therapeutic effect of lung adenocarcinoma.

However, there are some deficiencies in this study. First, the specific pathway of SPON2 activating CAFs and its relationship with FAP and α-SMA was not explored. Second, SPON2 expression was more downregulated after inhibiting lung adenocarcinoma exosome release than we expected, suggesting that other regulatory mechanisms derived from lung adenocarcinoma exosomes may exist.Third, due to limitation of time and funding, the role of SPON2 and corresponding mechanism have only been confirmed in cell experiments, and further verification in animal experiments is lacking. Nevertheless, this was the first and preliminary study to find the role and potential regulatory mechanism of SPON2 in CAFs in progression of lung adenocarcinoma.

## Conclusion

**Fig. 5:**
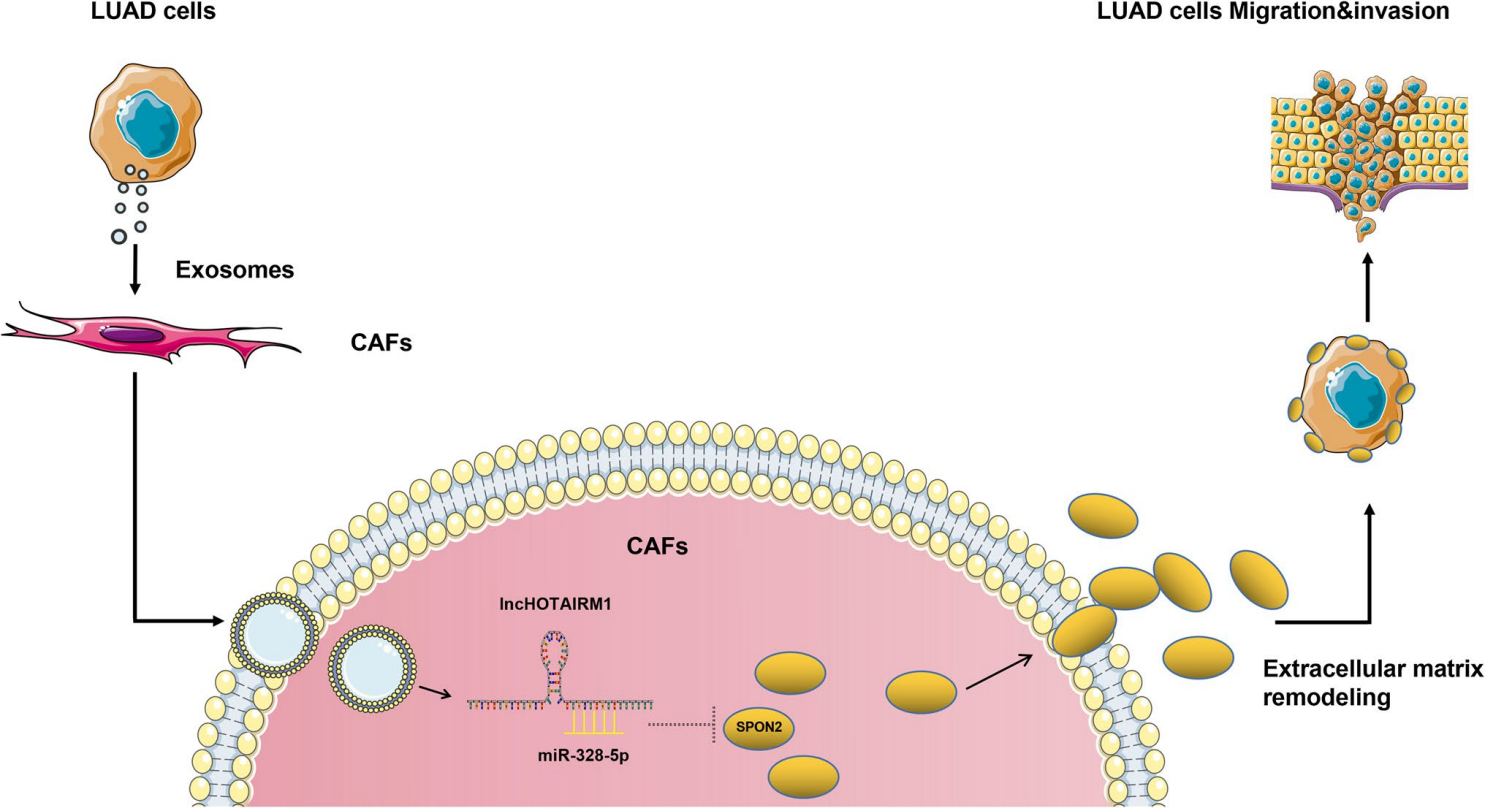
Schematic representation of a deduced regulatory network in which HOTAIRM1 regulates SPON2 through competitive adsorption of miR-328-5p

We studied the expression characteristics and biological function of SPON2 in lung adenocarcinoma, and confirmed that SPON2 is an extracellular matrix protein secreted by CAFs with high specific expression and promoting the invasion and metastasis of lung adenocarcinoma, and its expression level is significantly positively correlated with the degree of migration and invasion; SPON2 can enhance the promotion of CAFs on the invasion and progression of lung adenocarcinoma cells. Secondly, this study found that SPON2 can be derived from the exosome HOTAIRM1 of lung adenocarcinoma cells, which can affect the expression by competitive adsorption of miR-328-5p, so as to promote the invasion and metastasis of lung adenocarcinoma. Figure 5 is the schematic diagram of our mechanism.

## Supplementary Information

**Additional file 1**: Information About CAFs from Patients.

**Additional file 2**: Predicted miRNA binding to spon2 and HOTAIRM1.

**Additional file 3**:  lSupplementary Figure

## Data Availability

All data in our study were available from the TCGA database (http://cancergenome.nih.gov/).
