# Supplementary material for: Tumor-derived exosomal HOTAIRM1 regulates SPON2 in CAFs to promote progression of lung adenocarcinoma
Source: Discov Oncol. 2022 Sep 24;13:92. doi: 10.1007/s12672-022-00553-7 (PMC9509512; doi:10.1007/s12672-022-00553-7)
Supplement: Supplementary file 1 — Additional file 1: Information About CAFs from Patients. [file 12672_2022_553_MOESM1_ESM.docx]

| CAFs source patient information | |
| --- | --- |
| Tumor diameter |  |
| ≤1 | 4 |
| ＞1 | 6 |
| Age |  |
| ≤60 | 6 |
| >60 | 4 |
| Gender |  |
| Male | 5 |
| Female | 5 |
| Smoking |  |
| N/A | 8 |
| Y | 2 |
| Pleural Involvement |  |
| N/A | 8 |
| Y | 2 |
| Ki-67 |  |
| N/A | 7 |
| Y | 3 |
