# Supplementary material for: Tumor-derived exosomal HOTAIRM1 regulates SPON2 in CAFs to promote progression of lung adenocarcinoma
Source: Discov Oncol. 2022 Sep 24;13:92. doi: 10.1007/s12672-022-00553-7 (PMC9509512; doi:10.1007/s12672-022-00553-7)
Supplement: Supplementary file 3 — Additional file 3: lSupplementary Figure [file 12672_2022_553_MOESM3_ESM.docx]

**Supplementary materials**

**
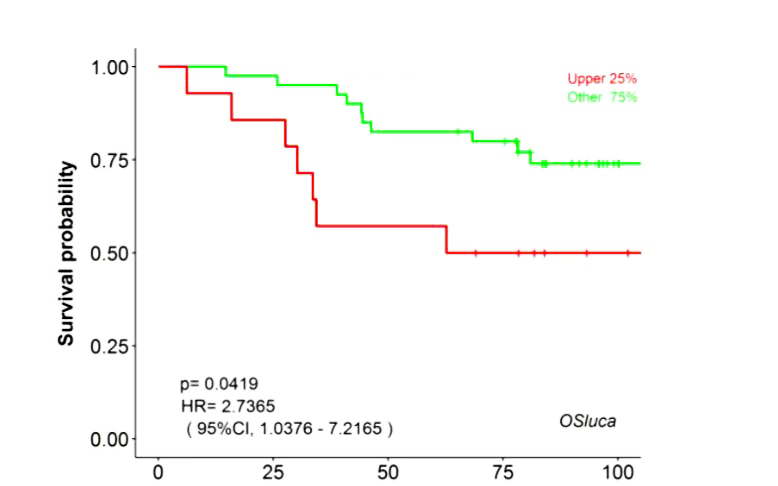
**

**Supplementary Figure 1. The GEO database analyzed the survival curve of high expression of SPON2 in T1 stage lung adenocarcinoma.**

**
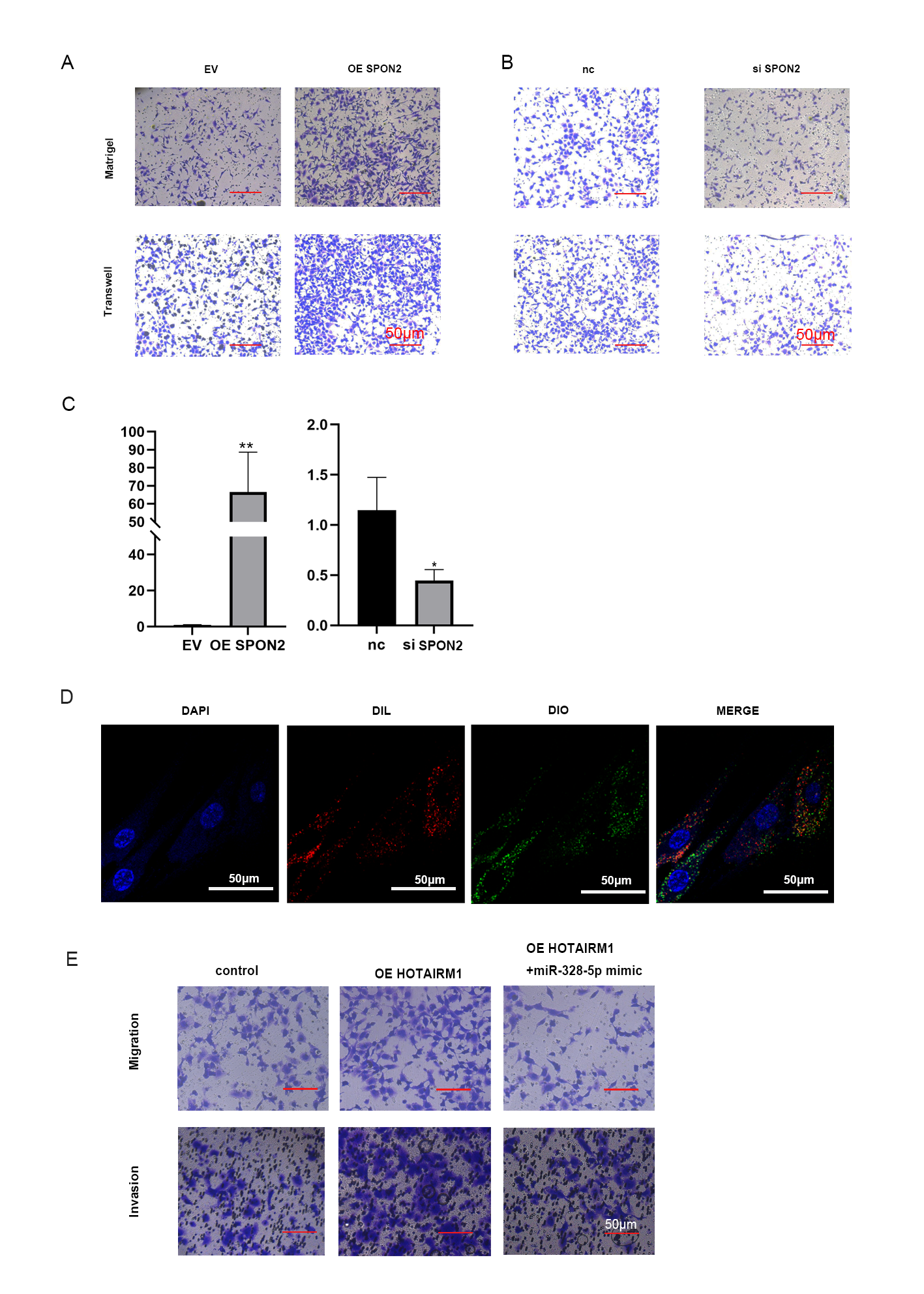
**

**Supplementary Figure 2.(A&B) The migration and invasion abilities were measured by transwell assay in H1299 cells (200× magnification). Scale bar =100 μm. (C) Efficiency of SPON2 knockdown and overexpression. (D) The fluorescence signal of CAFs cells labeled by DIL co-cultured with H1299 exosomes labeled by DIO was detected (400× magnification). Scale bar = 50 μm. (E) The migration and invasion abilities were measured by Transwell and Matrigel assay in H1299 cells (200× magnification). Scale bar = 50 μm.**

**
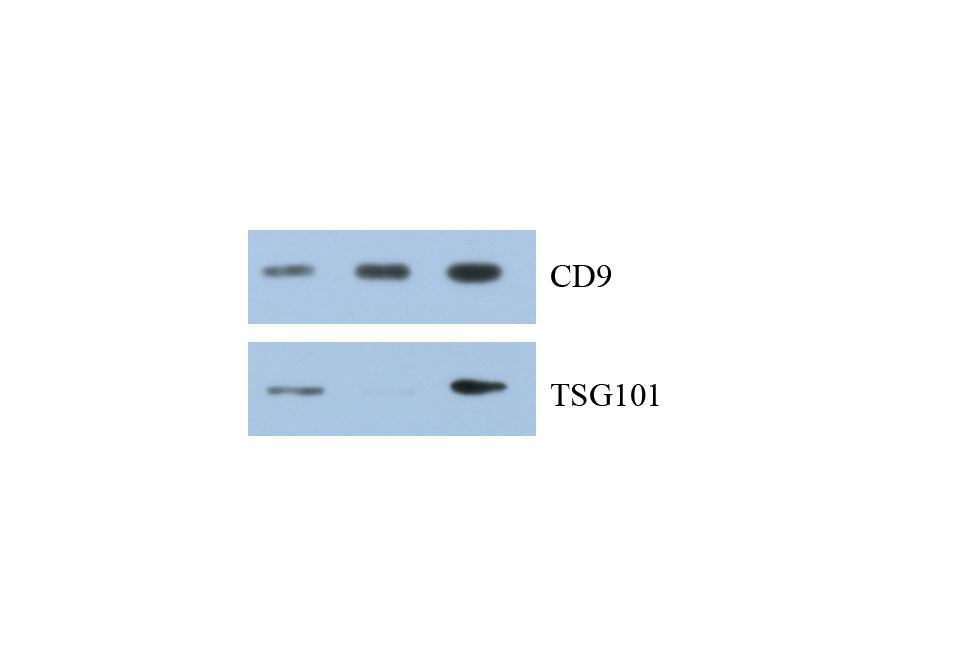
**

**Supplementary Figure 3. Identification of marker protein on the surface of exosome from A549 and H1299 by WB.**

**
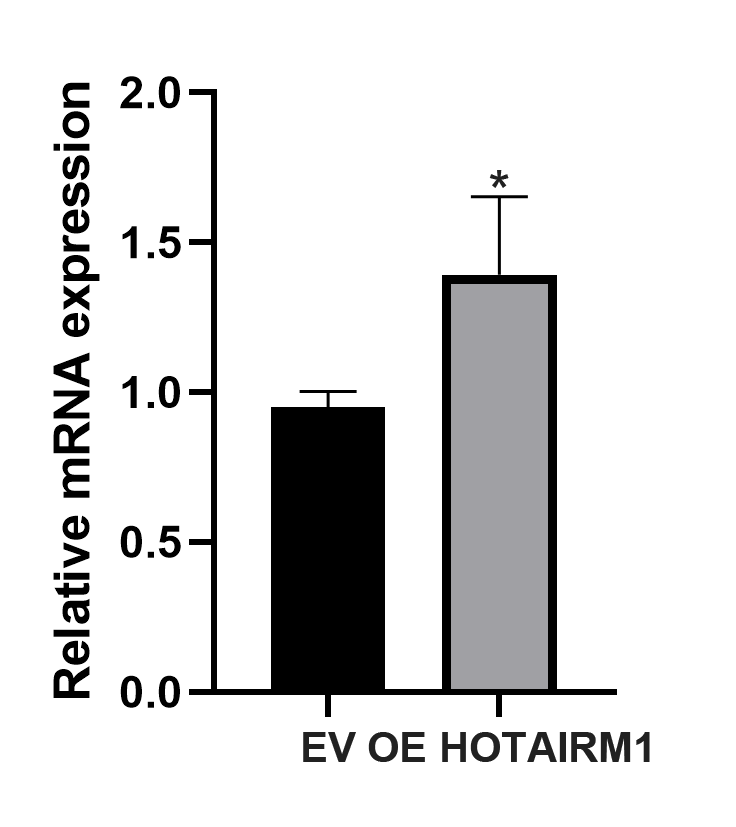
**

**Supplementary Figure 4. The expression of exosomal HOTAIRM1 was detected by qPCR after overexpression of HOTAIRM1 in A549 cell.**

**
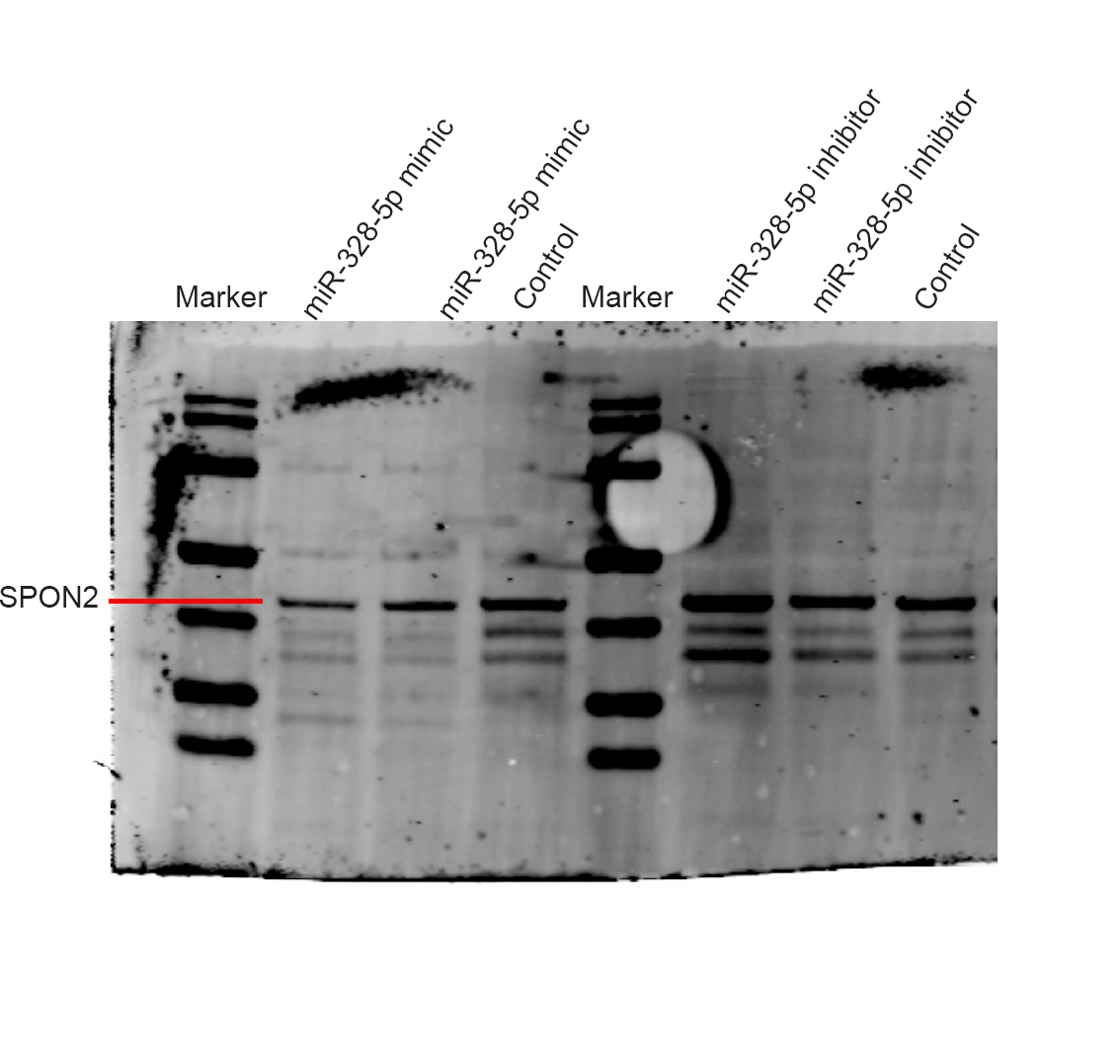
**

**Supplementary Figure 5. The protein levels of SPON2 regulated by miR-328-5p were detected by Western blot.**

**
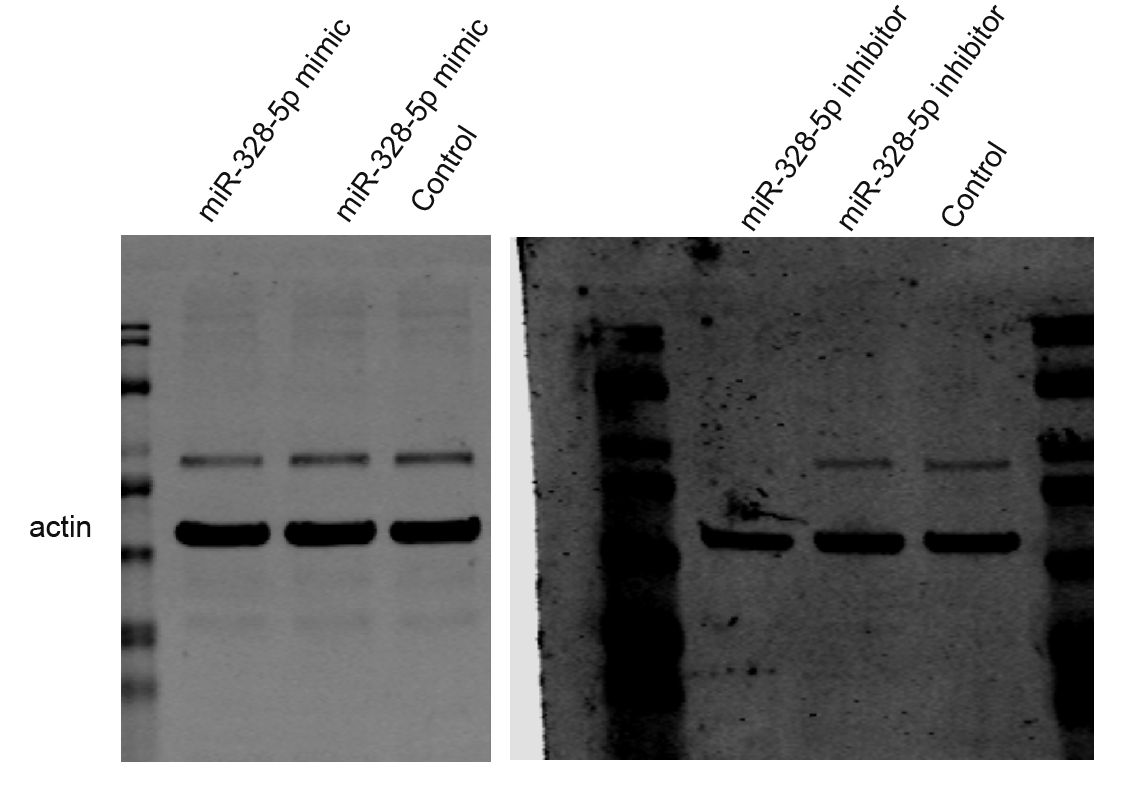
**

**Supplementary Figure 6. The protein levels of actin regulated by miR-328-5p were detected by Western blot.**

**
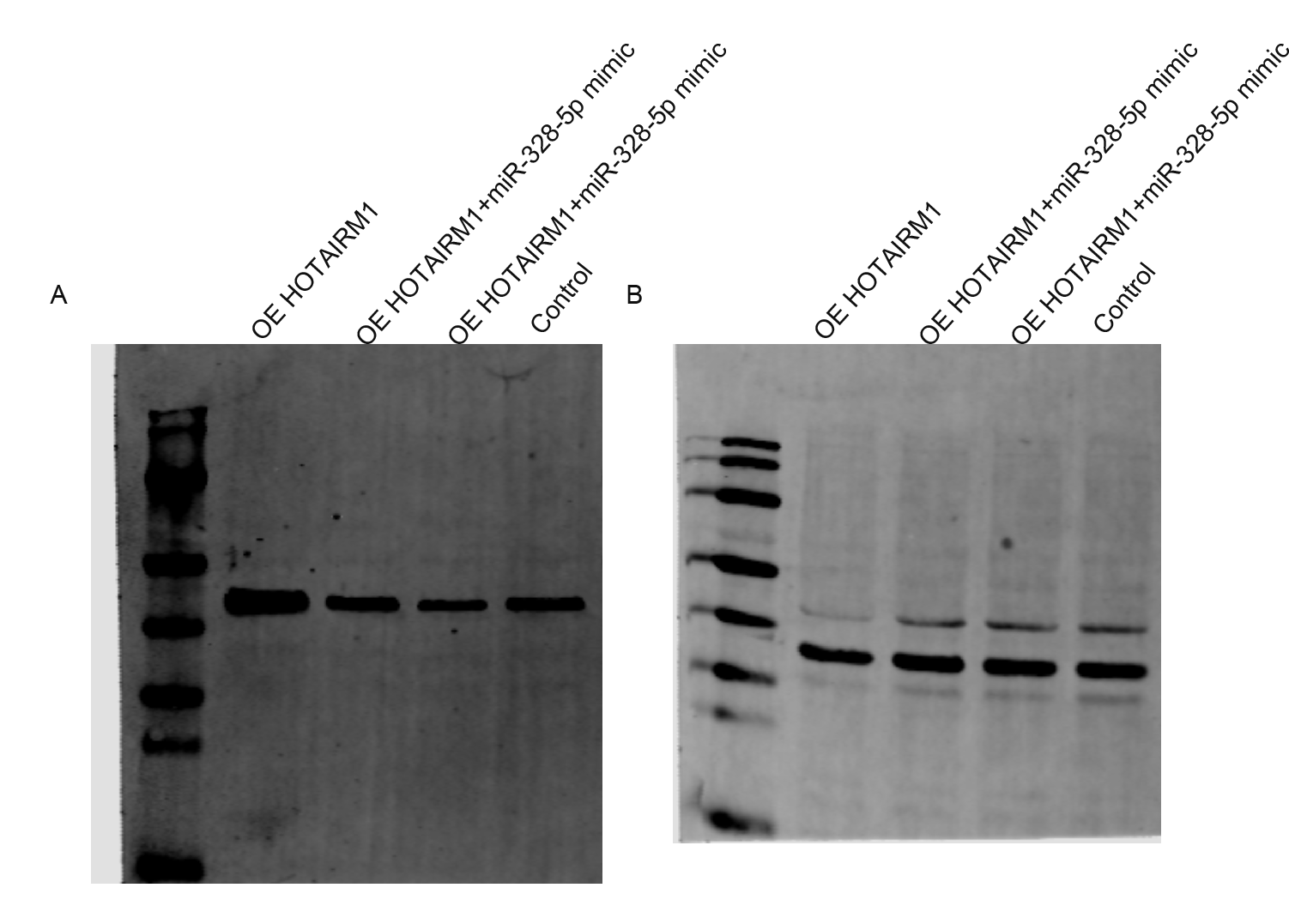
**

**Supplementary Figure 7. (A) The protein expression levels of SPON2 were analyzed by western blot in A549 cells.(B) The protein expression levels of actin were analyzed by western blot in A549 cells.**
